# Fracture identification and characteristics of carbonate underground gas storage: an example from the eastern area of Sulige gas field, ordos Basin, China

**DOI:** 10.1038/s41598-023-50072-w

**Published:** 2023-12-17

**Authors:** Jun Xie, Xiaofan Hao, Yuanpei Zhang, Jianguo Zhang, Yong Xia, Yilin He

**Affiliations:** 1 College of Earth Science and Engineering, Shandong University of Science and Technology, Qingdao, 266590 China; 2 Changqing Oilfield Exploration and Development Research Institute, Xi’an, 710012 China

**Keywords:** Geology, Petrology

## Abstract

The carbonate rock formations have obvious dual media characteristics, fracture development and good physical conditions, which are the main seepage channels and storage spaces for gas after the reconstruction of underground gas storage. The carbonate strata of the Ordovician system are important natural gas reservoirs in the eastern area of Sulige Gas Field in the Ordos Basin, and the identification and characterization of their fractures are of great significance for the modeling of fractures in the later stage and the improvement of the operation scheme of the gas storage. At present, there is little research on fractures, which restricts exploration and development. Therefore, taking the 39–61 gas storage reservoir in the eastern area of Sulige Gas Field in the Ordos Basin as the research object, this paper identifies and studies the characteristics of the fractures by core, microscopic, conventional logging curves, and imaging logging identification. The results show that the fracture length ranges from 5 to 15 cm and the width ranges from 0.1 to 3 mm. The fracture angles are mostly between 75° and 90° and the main direction is NW–SE. In conventional logging curves, porosity logging has a good response to fractures, while resistivity logging has a general response to fractures; In layers with more developed fractures, natural gamma values are mostly higher than 40API, rock volume density is less than 2.8 g/cm^3^, neutron porosity is greater than 12.5%, and acoustic time difference is greater than 160 μ s/m. This study is of great significance for improving the identification of carbonate fractures, enriching the relevant theories, and providing guidance for the construction of carbonate gas storage.

## Introduction

Carbonate reservoirs account for about 50% of global oil reserves^1,2^ and are a very important reservoir type. Underground gas storage is mainly used for gas peak shaving and safe gas supply, and is the infrastructure to ensure national energy security^3^. With the maturity of technology and theoretical knowledge, carbonate reservoirs have been developed into underground gas storage. Compared with sandstone gas storage, carbonate reservoir fractures are more developed and there is a great risk of leakage^4^. The generation and distribution of fractures in carbonate reservoirs are influenced by multiple factors, among which rock facies and structural changes play an important role^5^. Previous studies have shown that fractures not only provide pore space for fluid storage, but also greatly improve reservoir connectivity and permeability^6,7^, which also determines the production and exploration and development effect of oil and gas reservoirs, and the identification and characterization of carbonate reservoir fractures has always been the focus of scholars' research.

Fracture identification methods can be divided into direct methods (such as core observations, thin section and FE-SEM analysis, FMI logging) and indirect methods, which are carried out through the processing and analysis of conventional logging curves^8^. The conventional well logs include gamma ray (GR), spontaneous potential (SP), caliper (CAL), acoustic log (AC or DT), compensated neutron log (CNL), density (DEN) and resistivity (deep and shallow) logs^9,10^. Sun et al.^11^ and Lin et al.^12^ constructed characteristic parameters from conventional logging data to highlight the response characteristics of fractures and established a conventional logging fracture model^11^. He^13^ conducted systematic research on carbonate rocks respectively, and constructed a method for predicting fractures in reservoirs with different lithology using conventional logging data^13^. Chen and Wei^14^ proposed a conventional logging identification method for identifying fractures using dual lateral resistivity, acoustic transit time and compensated neutron intersection based on comparative core observation and imaging fracture feedback characteristics^14^. However, the responses of fractures in conventional well logs are weak and complex; this is especially true to wells low in fracture density^15^. It makes fracture identification through conventional well logs difficult. In view of the difficulty, a series of machine learning methods are introduced^16,17^, including adaptive neuro-fuzzy inference system (ANFIS) , GA-BP neural network^18^, Bayesian network theory and random forest and so on. There is also seismic fracture identification based on ResUNet and Dense CRF models^19^.

In the quantitative calculation of fractures, formation microimagers (FMIs) with high-resolution downhole images are widely used. Geng^20^ elaborated on the different characteristics of different fracture types in electrical imaging images^20^. FMI logging can perform subsurface fracture analysis and determine the direction of in-situ stress^1^. It is also used for reservoir description, particularly reservoir characterization^21^, by detecting fractures and other geological features or for physical property analysis^22^.The information in FMI logging can quantitatively determine the location, development degree and nature of fractures and then quantitatively obtain fracture parameters, laying a foundation for exploring the vertical and horizontal variation laws of filled fractures^23,24^.

Through literature research, it was found that research on the Sulige gas field mainly focuses on sedimentary facies, genesis, and reservoir formation mechanisms^25,26^, with limited research on reservoir fractures, resulting in some limitations in the construction of carbonate underground gas storage. Therefore, this article takes the 39–61 gas storage in the eastern area of Sulige Gas Field as an example, starting from the actual situation of the research area, adopts appropriate methods to analyse and evaluate it from multiple aspects, hoping to provide some assistance for the subsequent construction of gas storage.

## Regional geological overview

**Figure 1:**
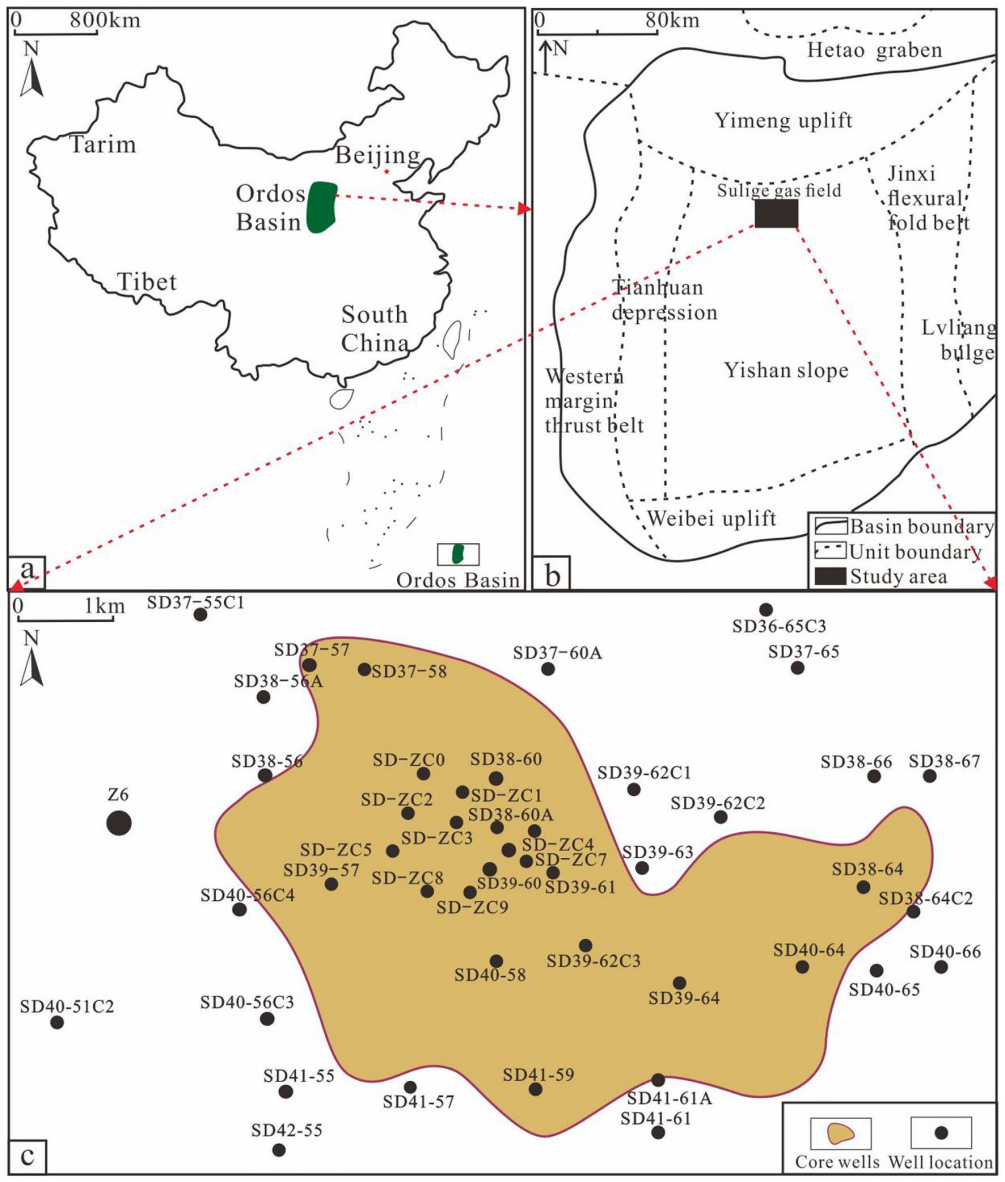
Location Map of the Study Area^30^. (**a**) Location Map of the Ordos Basin; (**b**) Structural Location Map of the Study Area; (**c**) Well Location Map of the Study Area. Fig (**a**) uses public data from the geospatial data cloud(https://www.gscloud.cn/search), the maps were generated using mapgis (Version 10.6.2.10, https://www.mapgis.com/).

The Ordos Basin is situated in the western part of the North China platform (Fig. 1a) and spans five provinces and regions, including Shanxi, Ningxia, Mongolia, Gansu and Shaanxi. The basin basement and the sediments of this layer exhibit a clear dual structure. It is a large multi-cycloratory craton basin with complex changes^27^. The Sulige Gas Field is located in the north-central part of the Ordos Basin and the eastern part of the Tianhuan Depression. Geographically, it is bounded by the Sulige Temple to the west, bordering the Etuoke Banner and the Jingbian Gas Field to the south (Fig. 1b). The SD39-61 gas storage is situated in the middle of the eastern region of the Sulige Gas Field (Fig. 1c). The Upper Paleozoic strata of the Sulige Gas Field consist of the Taiyuan Formation, Benxi Formation and Majiagou Formation, developed from top to bottom. The research horizon is located in the Mawu5 Submember of the Majiagou Formation of the Ordovician System. The sedimentary facies type is tidal flat facies, with a set of marine carbonate rock deposits predominantly dominated by dolomite and limestone^28^.

Owing to the influence of the sedimentary environment and diagenesis in both vertical and horizontal directions, the SD39-61 gas storage displays significant lithological differences between muddy limestone in the lower part and crystalline dolomite in the upper part. Dolomite is distributed in a lenticular shape on the profile and lithologically sealed by limestone on the lateral side. Therefore, it belongs to a typical lithological trap gas reservoir, exhibiting favourable reservoir formation conditions.

**Figure 2:**
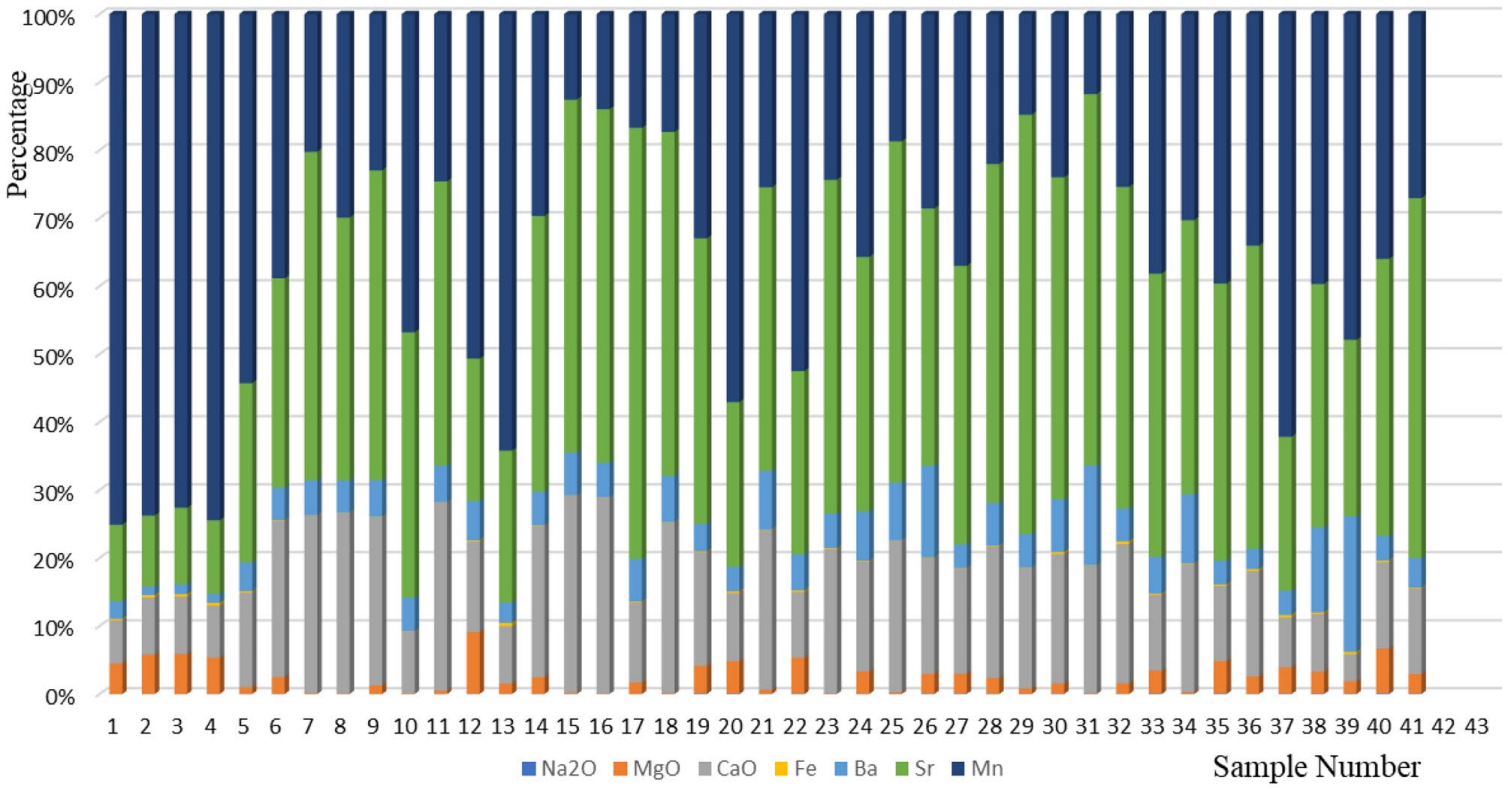
Percentage of trace element content.

This fracture study mainly focuses on sampling samples from the core wells and conducting trace element analysis based on relevant data. The trace elements in the study area mainly include Fe, Na, Mg, Ca, Ba, Mn, etc. The content of CaO and MgO is one of the indicators for determining rock type. Dolomite is a type of magnesian limestone that typically contains higher CaO and lower MgO. Limestone contains magnesium materials, minerals, or other magnesium compounds, while calcium materials and minerals are relatively small, resulting in lower CaO and higher MgO in the limestone. According to the percentage diagram of trace element content (Fig. 2), the CaO content in the samples is relatively high, while the MgO content is relatively low. The carbonate strata in the study area include limestone and dolomite, but this study mainly focuses on dolomite sedimentation.

## Materials and methods

### Materials

The original data used in this article were sourced from PetroChina Changqing Oilfield Company. The rock type and thickness data were obtained through statistical analysis of well cuttings description records based on the divided sub-layers. The core data for identification was obtained by photographing the core of the coring well and performing a detailed description, followed by statistical analysis of the developed fractures. The lithology and fracture filling of the sampled samples were obtained through casting thin section identification and based on the microscopic characteristics of the minerals. Microscopic data were then statistically summarised. The collected imaging logging data were processed using LEAD software; high-resolution borehole images were obtained through thin layer analysis, fracture identification and bedding analysis. Finally, the dip and fracture vectors were identified through manual interactive interpretation to obtain imaging logging maps.

### Methods

**Figure 3:**
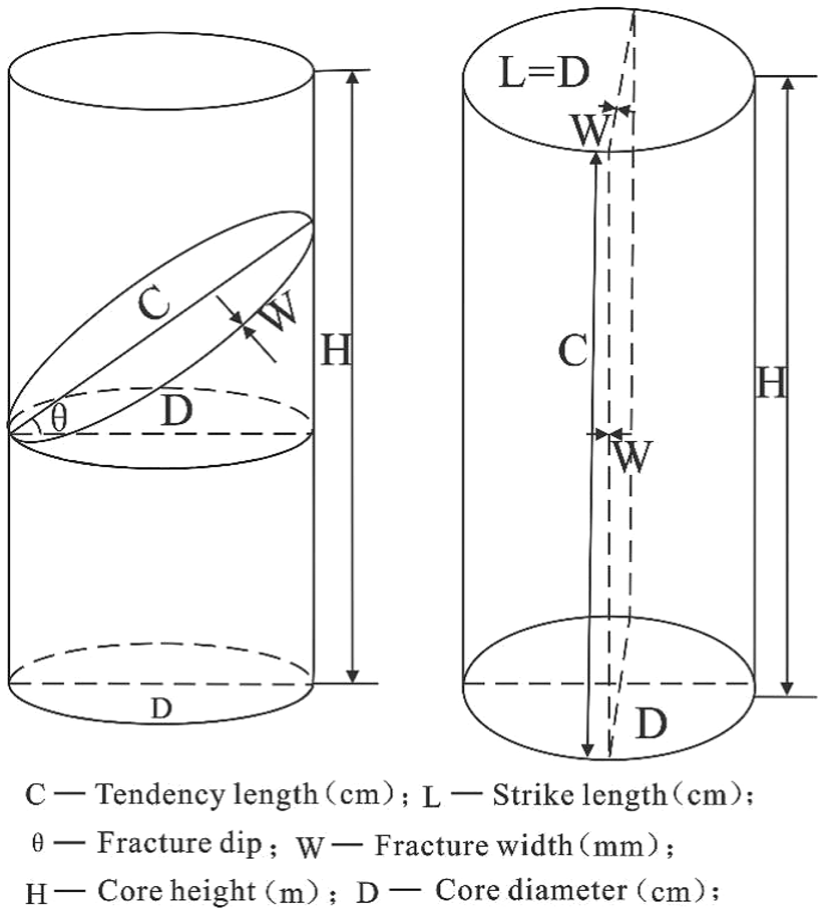
Characterisation of Core Identification Fracture Parameters.

This article used core identification, microscopic identification, conventional logging identification and electrical imaging logging identification methods to identify fractures. Core identification directly provided information on fracture length, width, dip angle and orientation. The interrelationship between various parameters was analysed through statistical analysis of core photos, and the development characteristics of fractures were studied from the core fracture observation (Fig. 3). Microscopic identification provided a more intuitive and detailed display of fractures through microscopic experiments, which allowed for observation of the development degree of fractures and determination of whether they were filled.

There are various conventional logging methods, and different logging methods have different sensitivities to fractures. The logging series that respond well to fractures are mainly divided into three categories: lithology logging, resistivity logging, and porosity logging. The conventional logging of lithological series mainly includes GR, SP, and CAL. The conventional logging of resistivity series generally refers to DLL (deep lateral and shallow lateral curves) and micro lateral (MLL). Bilateral resistivity logging adds shielding electrodes on the basis of ordinary resistivity, reducing the influence of mud on current diversion and surrounding rock, and improving the ability to vertically distinguish the formation. When fractures exist, the deep and shallow lateral resistivity values significantly decrease. When the fracture dip angle is small, there will be a "negative difference" phenomenon where the RLLD is smaller than the RLLS. When the fracture dip angle is large, there will be an opposite "positive difference" phenomenon. The conventional logging of porosity series mainly refers to CN, DEN, and AC. Conventional logging curves can have certain response characteristics to underground fractures (Table 1), which are the basis and foundation for identifying fractures using conventional logging.

**Table 1:** Responses of conventional logging for fractures.

Logging series	High-angle fractures	Low-angle fractures	Mesh fractures
DLL	Positive difference	Negative variance	Under the background of high resistivity, the reduction is relatively significant, with a certain thickness
AC	Not obvious	Jump wave	
CN	Responding but not obvious	Increase	Increase
DEN	No obvious	Obvious	Low anomaly
GR	Large attenuation amplitude	Small impact	Different degrees of attenuation
SP	Small impact	Large attenuation amplitude	Different degrees of attenuation
CAL	Expanding or shrinking	Expanding or shrinking	

Electrical imaging logging, also known as borehole wall micro-resistivity imaging, reflects changes in micro-resistivity caused by lithology or electrochemical heterogeneity in the formation opposite the electrode through the current intensity on the measuring instrument. After proper processing, it is scaled to a colour or greyscale image, which can reflect the underground structure. Static imaging, dynamic imaging and formation and fracture occurrence information in the log can help identify fractures. Static imaging is used to calibrate the colour code within the entire well section, reflecting the overall change characteristics and is suitable for macroscopic changes in formation conductivity. Dynamic imaging involves performing static colour calibration within a fixed window length to highlight local variation characteristics. Fractures appear as sinusoidal or cosine dark lines or strips in the imaging log.

In this study, rock samples from five coring wells at different depths, such as SD40-66, SD39-62C1 and SD37-58, were collected and subjected to microscopic experiments using optical or electron microscopy, including thin section analysis and scanning electron microscopy. However, owing to the limited number of coring wells and the information reflected, it is necessary to combine them with electrical imaging logging maps to accurately and comprehensively reflect fracture information. Using a rose diagram to represent the characteristics of fractures, the relationship between the strike of the fractures and the direction of the maximum principal stress can be obtained. The filling degree and dip angle of the fracture can predict whether the location has conditions for gas reservoir communication and migration.

## Fracture identification

Various types of fracture systems have been formed in the Mawu 5 member of the Majiagou Formation under the weathering, denudation and chemical dissolution of surface water or groundwater. These fractures provide a foundation for the migration and storage of natural gas. Based on the existing data statistics in the study area, fractures in the study area were identified through core identification, microscopic identification and imaging logging identification methods.

### Core identification

**Figure 4:**
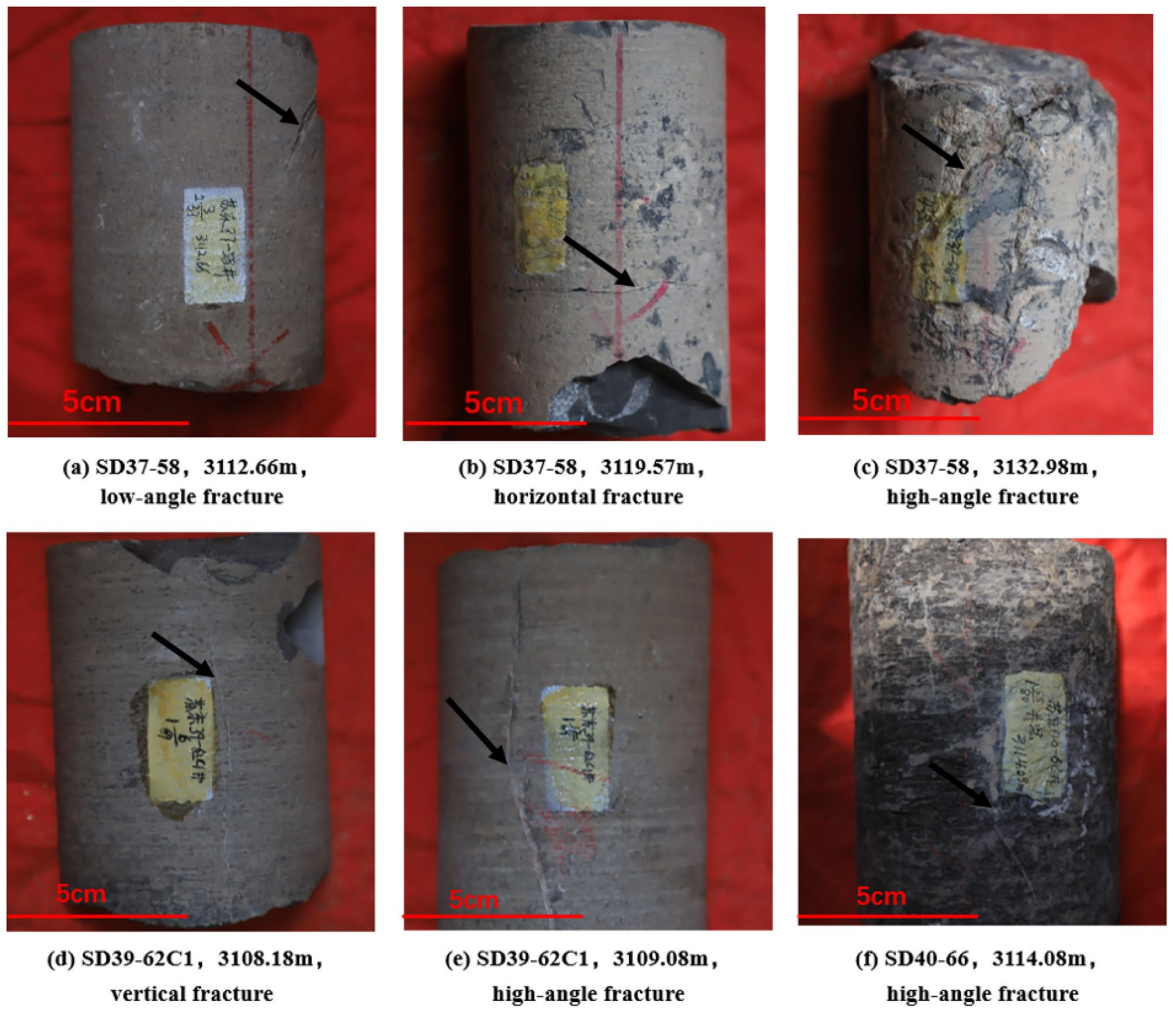
Partial Fractured Core Identification Results. (**a**) SD37-58, 3112.66 m, medium crystalline dolomite, with low angle fractures developed; (**b**) SD37-58, 119.57 m, microcrystalline limestone, with horizontal fractures developed; (**c**) SD37-58, 32.98 m, microcrystalline limestone develops high-angle fractures; (**d**) SD39-62C1, 3108.18 m, calcareous dolomite, with vertical fractures developed; (**e**) SD39-62C1, 3109.08 m, dolomite, with multiple high-angle fractures developed; (**f**) SD40-66, 3114.08 m, limestone, high-angle fractures developed.

Core identification is an intuitive and objective method for reflecting underground fracture information and has positive significance for subsequent fracture description and evaluation. For example, in the SD39-61 gas storage, six cored wells and three partial fractures have been core identified (Fig. 4). Through statistical analysis of the fractures in the core photos of six wells, it was found that the fracture development in the study area is inconsistent. In general, the injection production well area, i.e., the core area, has developed fractures, while wells SD37-58 and SD39-64 have generally developed fractures. The fracture length is between 5 and 15 cm; the width is between 0.1 and 3 mm; and horizontal fractures, high-angle fractures and vertical fractures are all developed in the study area.

### Microscopic identification

Microscopic identification mainly involves using a microscope to observe ordinary thin slices, morphology, width, length, fracture surface, corrosion and filling of fractures and statistically describing the fractures that exist in rocks. The first type of fractures observed is structural fractures and pressure solution fractures. Structural fractures are various displacements or fractures caused by external forces in the rock mass, generally forming an angle with the bedding surface, characterised by straightness, smooth walls, long extension and regularity. The suture has a clear relationship between cutting or dissolving fossils or debris and has suture columns of varying heights. The intersection angle with the layer is greater than 30°^29^.

**Figure 5:**
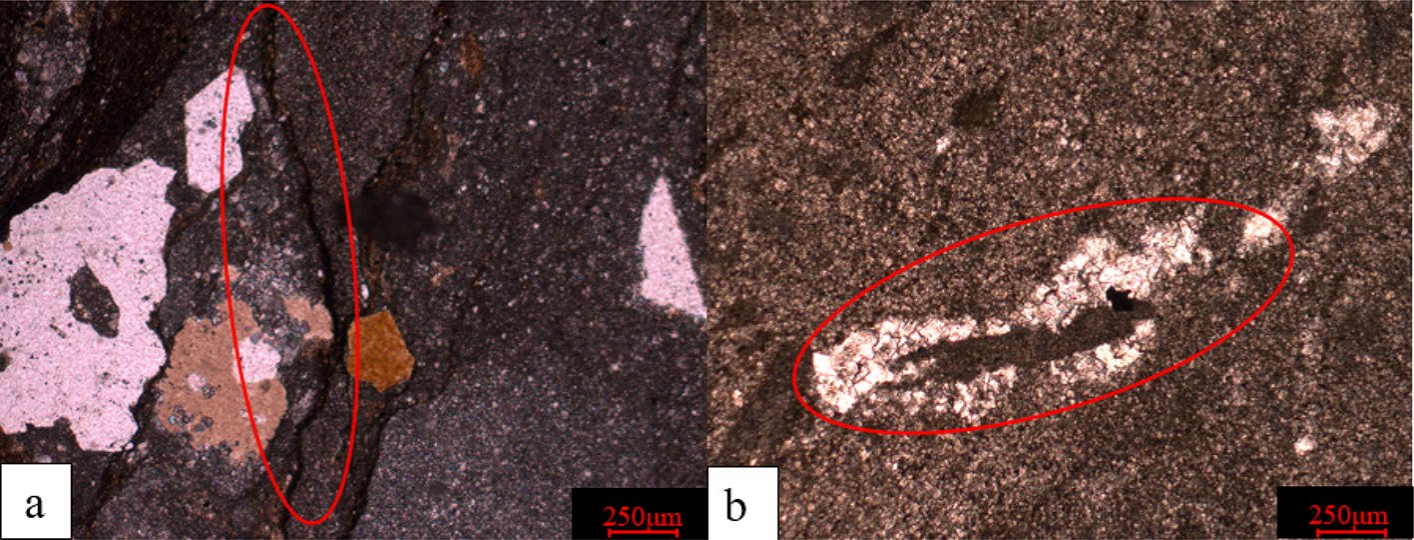
Schematic Diagram of Identifying Fracture Characteristics under a Microscope. (**a**) SD39-62C1, 3140.51 m, containing fine-grained limestone in the cloud, with multiple unfilled fractures developed, × 124 (+); (**b**) SD-ZC7 3115.55 m, silty fine crystalline dolomite, developed fractures, filled with bright calcite, × 124 (+).

Next is the degree of filling: an unfilled fracture has only two walls and no other minerals (Fig. 5a). If the fracture is semi-filled and not fully crystallised, it has extremely good crystals. If the filling is dissolved after deposition, the wall of the solution pore often presents irregular edges. The determination of fracture-filling minerals can be based on the microscopic characteristics of the minerals. For example, calcite exhibits granular, colourless and flashing characteristics under a single polariser; under an orthogonal polariser, it shows an advanced white colour, with symmetrical extinction (Fig. 5b). Pyrite is homogeneous and black under orthogonal polarisers.

A total of four wells in the study area were observed under a microscope. During fracture identification, statistics were made on the classification of fractures and the filling degree of fractures. Additionally, a statistical table of effective fractures identified by thin slices was prepared (Table 2).

**Table 2:** Statistical table of effective fractures identified by thin slices.

Well	Structural fracture (article)	Dissolution fracture (article)	Stylolite fracture (article)
SD39-62C1	7	26	3
SD40-66	0	1	0
SD37-58	0	3	0
SD39-57	0	8	0

### Identification of fractures by conventional logging

From four core wells in the study area, 16 non fractured sections with less developed fractures (fracture surface density < 1.5 cm/cm^2^) and 23 fracture sections with more developed fractures (fracture surface density > 1.5 cm/cm^2^) were selected. The conventional logging response characteristics of these sections were studied through on-site core observation. After analyzing the fracture responses of various logging sequences, 7 logging sequences (DEN, AC, CNL, RLLS, GR, CAL, SP) were selected to obtain conventional logging parameters for both fractured and non-fractured sections.

**Figure 6:**
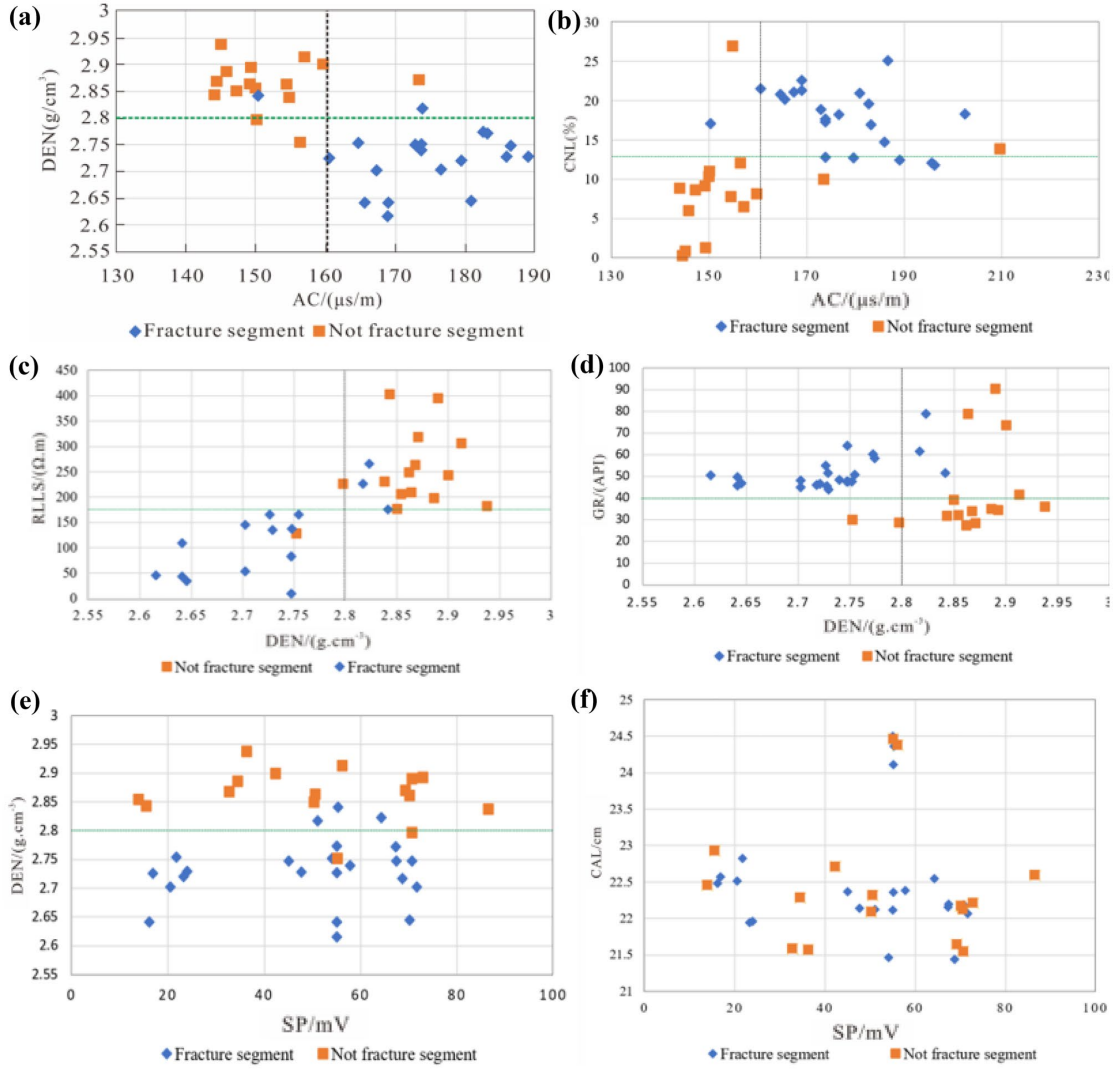
Cross plot of conventional logging sequences in the research area. (**a**) intersection diagram of DEN and AC; (**b**) intersection diagram of CNL and AC; (**c**) intersection diagram of RLLS and DEN; (**d**) intersection diagram of GR and DEN; (**e**) intersection diagram of DEN and SP; (**f**) intersection diagram of CAL and SP.

The intersection analysis of the main conventional logging sequences between the fractured and non fractured sections (Fig. 6) shows that the fractured sections in the study area exhibit the following characteristics: ① high GR values, with no significant differences between SP and CAL and non fractured sections (Fig. 6d–f); ② RLLS is lower than the non fractureed section (Fig. 6c); ③ The DEN value is smaller than the non fractureed section, and the AC and CNL values are larger than the non-fractureed section (Fig. 6a–c).

From the logging responses of fractured and non fractured sections in the study area, it can be seen that porosity series logging has a good overall response to fractures; due to the large variation range of fracture dip angle in the study area, the difference in deep and shallow lateral resistivity values is relatively small, and the response of resistivity series logging to fractures is average; the lithology series logging has the worst response to fractures, and analysis suggests that the study area has frequent thin interbeds. The impact of lithology on natural potential and wellbore diameter exceeds that caused by fractures. Through intersection analysis, combined with conventional logging sequence parameters of fractured and non fractured sections, several parameter characteristics that have a more obvious response to fractured sections were obtained (Table 3).

**Table 3:** Parameter characteristics of fracture segments in conventional logging sequences in the study area.

Classification	DEN/(g cm^−3^)	AC/(μs m^−1^)	CNL/%	GR/(API)	RLLS/(Ω.m)
Fracture segments	< 2.8	> 160	> 12.5	> 40	< 175
Not fracture segments	> 2.8	< 160	< 12.5	< 40	> 175

Based on the response characteristics of conventional logging curves to fracture segments, select the acoustic time difference (AC) curve with good response effect for single curve analysis:

The increase in the amplitude of the acoustic time difference or the small oscillation of the curve can be used as identification indicators for low angle and horizontal fractures. When large-scale horizontal and low angle fractures are developed, the sound wave propagation path is orthogonal to it, and the time difference curve has a unique cycle jumping feature, with a small serrated curve. In this study, when the AC curve shows abnormally high values or there is no significant change in jumping changes, it is judged as a fracture development segment.

**Figure 7:**
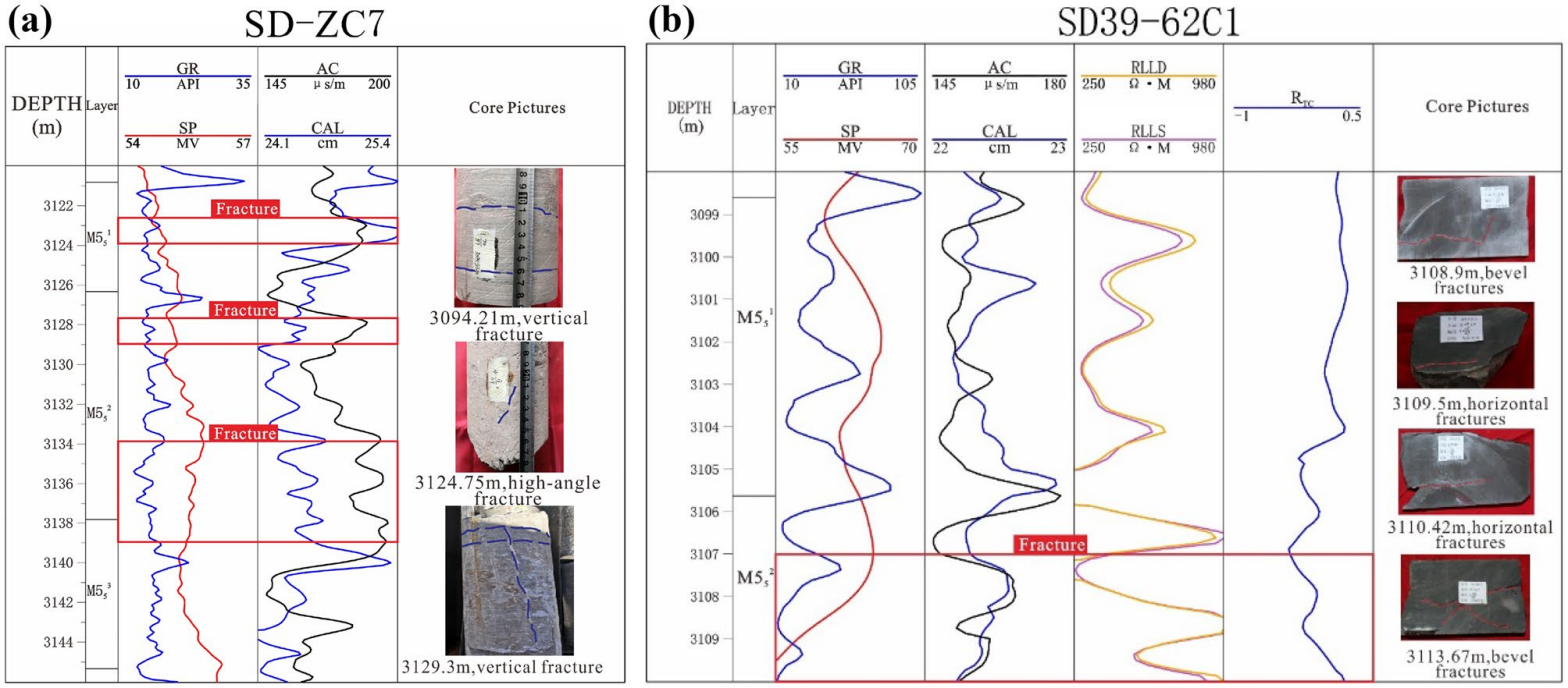
Identification results of conventional logging curves (**a**) identification results of AC curve in SD-ZC7; (**b**) identification results of deep shallow resistivity difference ratio method for well SD39-62C1.

The average AC curve of this well section is 180.025 μs/m. The abnormal high values are mainly distributed in the 3123–3124.5 m, 3128–3129 m, and 3134-3138 m sections (Fig. 7a). After comparison with core observation, it was found that the recognition accuracy is around 40%. Overall, the identification results of acoustic time difference logging are generally consistent with the core observation results.

Due to the limitations of a single logging curve in identifying fractures, it is generally consistent with actual fracture development; Based on previous research findings, it has been found that the deep shallow lateral resistivity curve, after a series of calculations, can help better identify fractures and achieve good response characteristics. Therefore, on the basis of a single curve, this study also used bilateral resistivity curves to analyse fracture development and verify its accuracy in combination with core identification.

On the fracture development profile, there is a certain amplitude difference between the deep resistivity curve and the shallow resistivity curve, but this amplitude difference is generally very weak and can only qualitatively describe the fracture. In order to expand the amplitude difference, the resistivity invasion correction difference ratio method is used to describe the fractures. Firstly, calculate the true resistance of the formation after invasion correction, and combine it with the actual measured deep and shallow lateral resistivity to calculate the difference ratio after invasion correction. The calculation formula is as follows:

1 $$R_{{\text{T}}} = {2}.{\text{589R}}_{{{\text{LLD}}}} - {1}.{\text{589R}}_{{{\text{LLS}}}}$$

2 $$R_{TC} = \left( {R_{T} {-}R_{LLS} } \right)/R_{LLS}$$

Among them: *R*_T_ is the ratio of deep to shallow resistivity difference, R_LLD_ is the deep lateral resistivity of the formation, R_LLS_ is the shallow lateral resistivity of the formation, and R_T_ is the true resistivity of the formation corrected for invasion. When the local layer is a fractured gas layer, *R*_T_ > R_LLS_, ***R***_***TC***_ > 0; When the local layer is a fractured water layer or a dense formation, *R*_T_≈ R_LLS_, ***R***_***TC***_ = 0.

In oil field development, fracture development sections are determined based on logging data, visual logging, core observation, and other methods, and compared with the resistivity difference ratio to comprehensively determine the resistivity difference ratio threshold for the fracture development section in the analyzed oil reservoir.

Due to the significant differences in resistivity caused by lithology, in order to better observe the relationship between dolomite and fracture development, the 3098.00–3115.00 m dolomite section of SD39-62C1 well was selected for fracture logging response analysis, and the value of *RTC* was calculated using conventional logging data. According to the identification results of the logging curve, the 3107–3110 m section is the predicted fracture development section. Comparing the core photos of this section with the RTC value, the comprehensive results show that the fracture identification at the RTC response is accurate (Fig. 7b). And when the RTC curve value is above 0.2, it can be considered as a fractured well section.

### Identification of fractures by imaging logging

Imaging logging is characterised by high resolution and full borehole scanning, which can determine the dip angle, dip, structural characteristics, geometric morphology and degree of fracture development of the formation. Owing to the abundance of information reflected by imaging logging, identifying fractures using these data requires differentiating between the formation mechanisms and characteristics of various geological structures on the logging map.

A single fracture is typically distributed in the form of a sinusoidal curve on the imaging map, and the amplitude of the curve can reflect the magnitude of the fracture dip angle. The lithology on both sides of the fracture sine curve is continuous, allowing for differentiation from bedding planes or other structures. As a result, carbonate rocks are highlighted in the electrical imaging static image. Unfilled fractures exhibit a linear characteristic of black lines in the electrical imaging log, whereas filled fractures show varying colours from deep to shallow. Fractures filled with argillaceous or pyrite minerals exhibit a dark colour, while calcite filling exhibits a high-resistance characteristic of white. Therefore, identifying whether the formation fractures are filled requires a comprehensive study combined with core calibration information.

**Figure 8:**
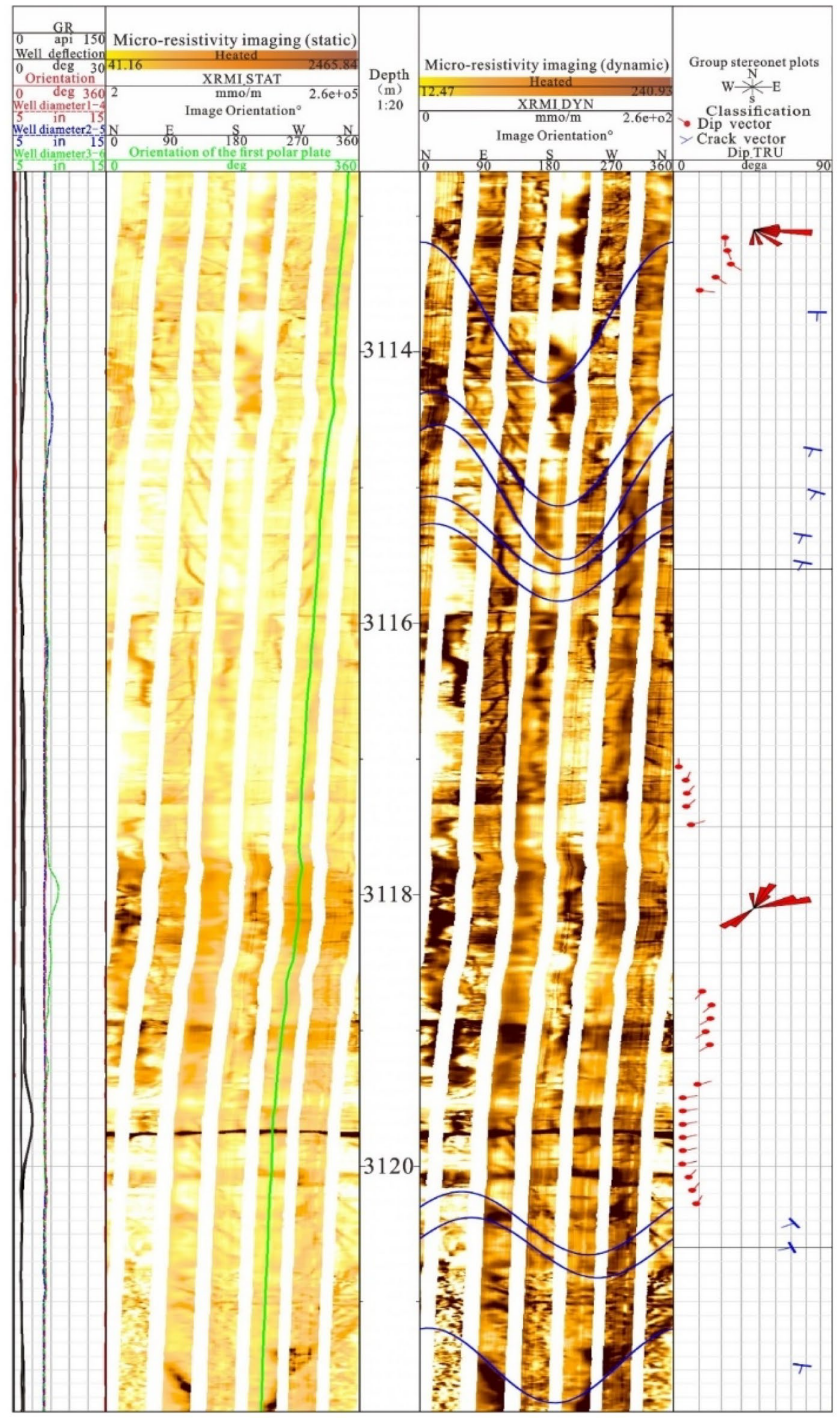
Characteristics of SD39-64 3112–3122 m imaging logging.

In the study area, imaging logging data have been collected from three wells, with well SD39-64 serving as an example (Fig. 8). The identified well section of well SD39-64 is 3112–3122 m, and the upper and lower parts of the electrical imaging static image are relatively bright, indicating higher resistivity in the middle part compared to the upper and lower strata. From the dynamic image, fractures are highly developed and dense, while dissolved pores in the layer are relatively developed. Based on the high amplitude characteristics of the sinusoidal curve and the information displayed by the fracture vector, it is found that high-angle fractures are primarily present. The inclination vector of the stratum indicates that the inclination amplitude of the stratum is relatively small, averaging approximately 10°. Through research, the identification results of electrical imaging logging are relatively consistent with the identification results of core photos and micro fractures.

**Figure 9:**
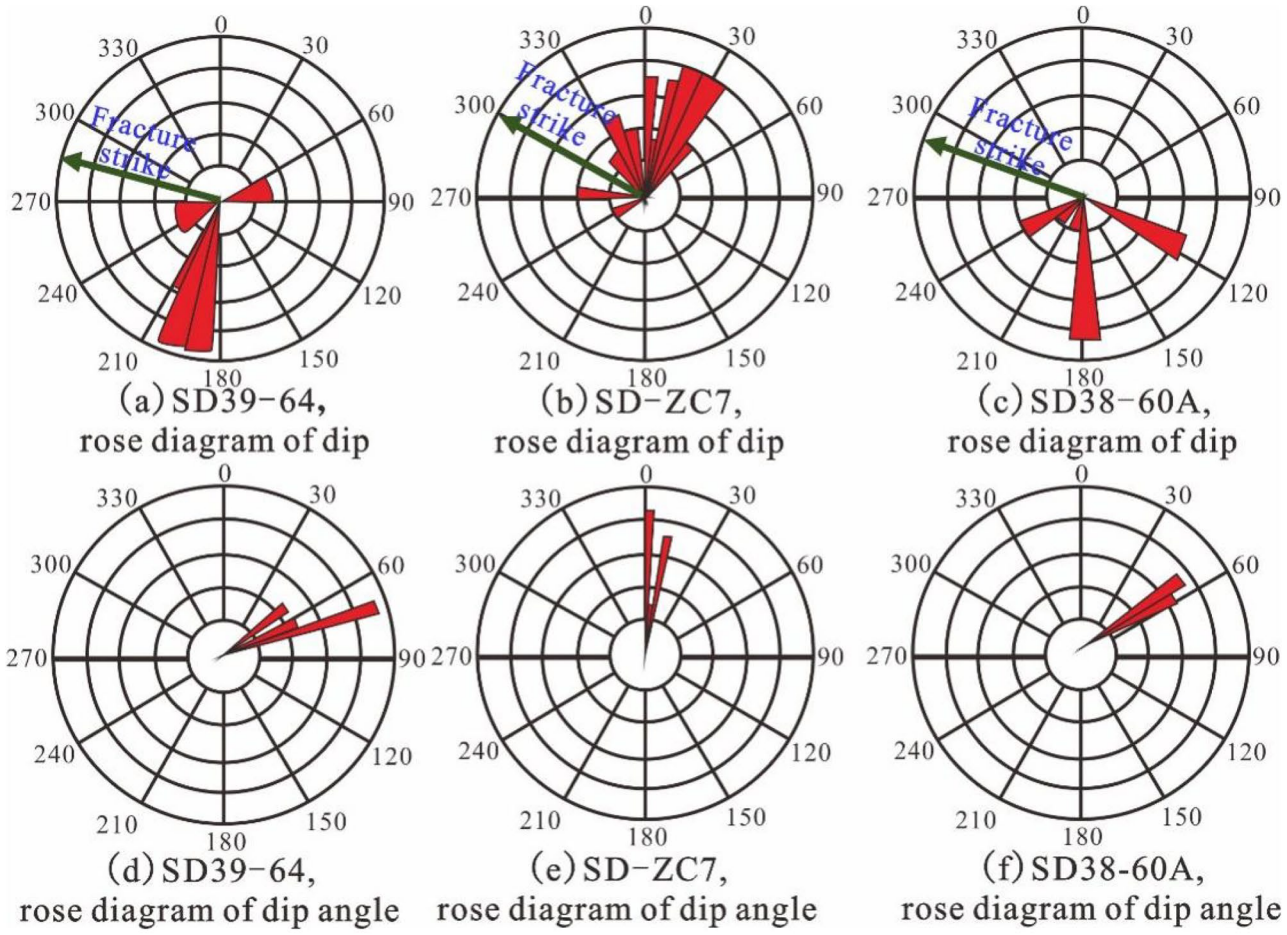
Rose diagram of SD 39–61 gas storage fracture dip angle (Inclination).

The electrical imaging log can provide an intuitive reflection of the occurrence of strata and fractures. Conducting statistics on the fracture vector column involves using the circumference (radius) length to represent the tendency (inclination) and a certain line length to represent the number of fractures. Using this data, a tendency (inclination) rose diagram can be drawn. The dip direction of SD39-64 is between 180°-210° (Fig. 9a), and the dip angle is concentrated around 75° (Fig. 9d); the dip direction of SD-ZC7 is concentrated at 20°-35°(Fig. 9b), the inclination is relatively small, and the dip angle is about 5° (Fig. 9e); SD38-60A tends to be around 180° (Fig. 9c) with dip angle close to 60° (Fig. 9f). Overall fracture extension direction (strike) is predominantly northwest-southeast, with most fractures falling between 75° and 90°, appearing as high-angle fractures.

### Fracture description

The above methods were used to statistically analyse the characteristic parameters, such as the number, length, width, filling condition, strike and dip angle of fractures in certain wells located in SD39-61 gas storage.

The identification results indicate that the study area is primarily characterised by high-angle and vertical fractures, with filling materials consisting of calcite, argillaceous and pyrite. Horizontal and vertical joints are filled with argillaceous and calcite, while high-angle fractures are mostly unfilled or partially filled. Core data and microscopic observation revealed that most fractures had limited extension length and small opening degrees, leading to the conclusion that the study area is primarily composed of small and micro fractures, based on relevant standards. Through the combination of logging data, rock anisotropy and macroscopic fracture occurrence information, the primary direction of fracture is determined to be in the NW–SE direction.

## Discussion

**Figure 10:**
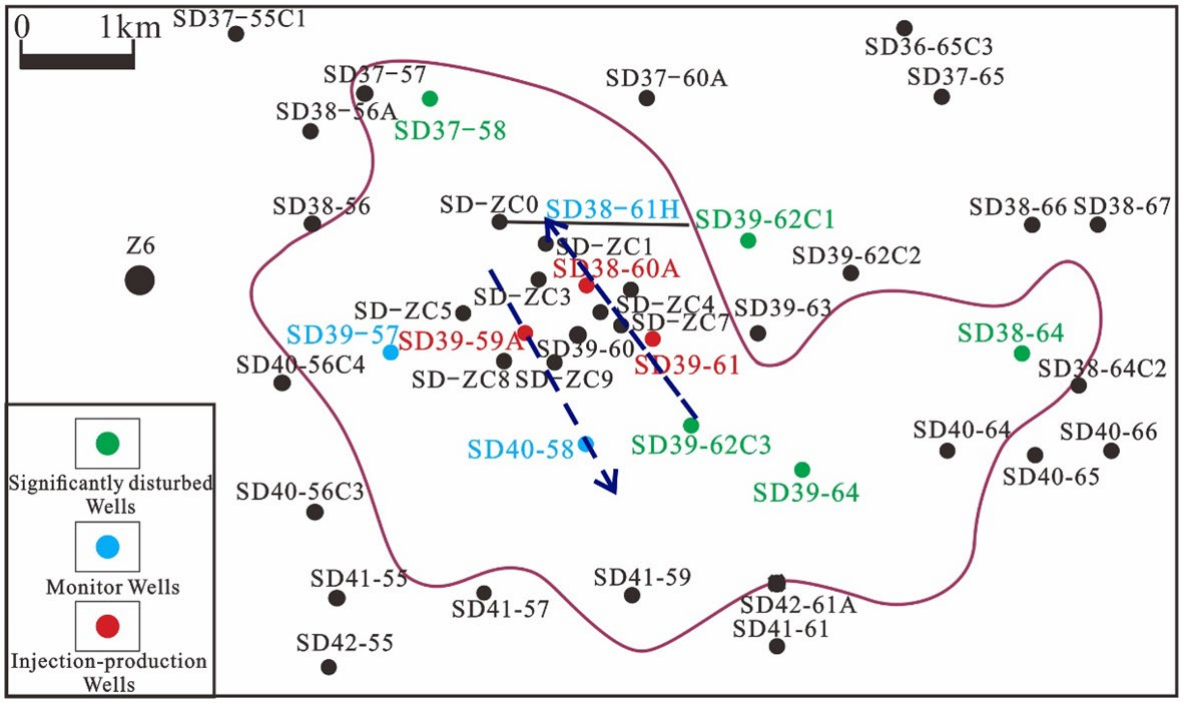
Effect diagram of gas injection.

Observation of atmospheric pressure changes in surrounding wells through interference testing can reflect reservoir connectivity and verify the accuracy of fracture identification. Gas injection work was conducted on three injection and production wells (SD39-59A, SD38-60A and SD39-61) in the study area, and pressure changes in the nine monitoring wells around them were observed. Based on the gas injection monitoring results (Fig. 10), wells with significant interference from gas injection were primarily located in the core area, resulting in formation pressure changes for SD38-61H, SD39-57 and SD40-58. Monitoring logs for the same layer (SD37-58, SD38-64, SD39-64 and SD39-62C1) in the peripheral area were not significantly disturbed. Overall, the core area displayed good connectivity, while the peripheral area had poor connectivity. The affected direction was predominantly in the NW–SE direction.

**Figure 11:**
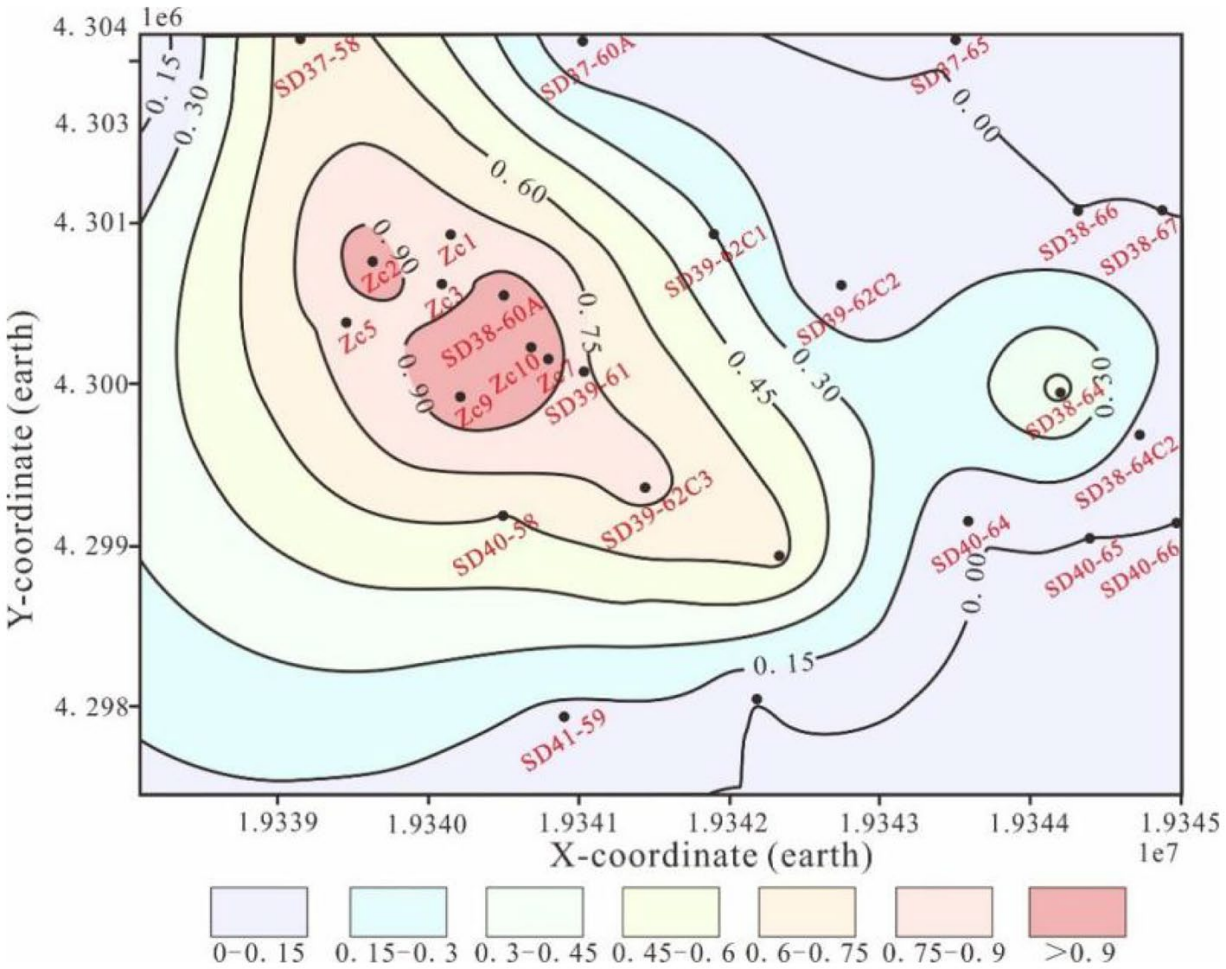
Fracture density distribution diagram.

Coherent cube is a seismic attribute developed in the 1990s, which has become a commonly used technique to highlight discontinuities. It is a method that uses the similarity between adjacent seismic waveforms to detect geological anomalies, such as fracture development zones, resulting from abnormal bodies like abrupt lithological changes and fractures. The technology detects differences in kinematic and dynamic characteristics between reflected waves of adjacent seismic channels. By digitising the seismic fracture coherence volume map in the collected data and using interpolation functions, the coherence volume distribution map of the entire region can be obtained, providing a fracture density distribution map. The darker the colour, the denser the fractures, with the overall trend extending from northwest to southeast (Fig. 11). The core of the study area appears darker and denser, while in the peripheral well area, the colour is lighter, and the density is relatively small.

Comparing the fracture effect direction observed from the injection production effect map and the relatively developed fracture position observed from the density distribution map with the fracture identification results in the previous article, the results are relatively consistent. The comprehensive identification method using multiple means such as core, mirror and electrical imaging logging is relatively accurate for fracture identification and can identify fracture density, length, opening, dip angle and filling conditions. Compared with a single method for identifying fractures, this method is more comprehensive, specific and persuasive. However, the study has limitations owing to limited data, resulting in only partial data being subjected to corresponding descriptive statistics.

Recent exploration results indicate that the Lower Paleozoic gas reservoirs in eastern Jiangsu have enormous exploration and development potential. The SD39-61 gas storage well area is rich in oil and gas resources, with an advantage in constructing underground gas storage. However, the complex surface and underground conditions, as well as the constraints of reservoir fracture development, have posed difficulties for the reconstruction of underground gas storage. After several weeks of injection production experiments, drilling fluid leakage occurred. By identifying and describing fractures in this study, and analysing parameters such as the spatial distribution characteristics and primary directions of fractures, the impact of fracture distribution on the injection and production of gas reservoirs can be clarified, so that on the basis of the existing scheme, the well position can be adjusted at a certain angle to the fracture distribution direction, so as to increase the injection efficiency and control the edge injection and production. There are many methods for identifying fractures in carbonate gas reservoirs, and the selection of specific methods should be based on the actual geological situation, starting with the collected data and selecting suitable and effective methods for fracture identification.

## Conclusion

1. Based on data from coring wells, this study employed core identification, microscopic identification under the microscope and imaging logging identification methods to identify fractures caused by physical stress and chemical dissolution in the study area. The analysis and evaluation were conducted on fracture length, orientation and filling materials. The identification results indicated that the fracture length in the study area ranges between 5 and 15 cm, with a width between 0.1 and 3 mm. Regarding occurrence, fractures primarily develop as high-angle and vertical fractures, with fracture angles predominantly between 75° and 90°. Genesis is mainly attributed to structural fractures, dissolution fractures and diagenetic fractures; most fractures are filled with argillaceous and calcite. The fracture tendency is mostly between 240° and 250°, and the extension direction and maximum principal stress direction of the fracture are from northwest to southeast.
2. Core identification can intuitively reflect underground fracture information; Microscopic identification can observe the morphology, width, length, corrosion, and filling of fractures; But the core and thin section data are not comprehensive. Imaging logging has high resolution, which can accurately calculate the occurrence of fractures and quantitatively evaluate various parameters of fractures. It is relatively accurate in identifying formation fractures; However, the imaging logging method has a higher cost.
3. The porosity series logging methods in conventional logging curves have the most obvious response to reservoir fracture identification. The parameter characteristics of each logging curve are: the value of GR is mostly higher than 40 API, rock volume density is less than 2.8 g/cm^3^, neutron porosity is greater than 12.5%, and acoustic time difference is greater than 160 μ s/m; The resistivity series logging response takes second place, and the deep shallow lateral resistivity difference ratio method has a high degree of agreement with actual results, which is of great significance for logging identification and quantitative evaluation of reservoir fractures. Compared with other methods, conventional logging identification is an effective and economical method. Therefore, in practical research work, conventional logging curves can be used to identify fractures in carbonate reservoirs.

## Data Availability

The datasets generated and/or analyzed during the current study are not publicly available but are available from the corresponding author on a reasonable request.

## References

[1] Mojtaba R, Shahram S, Bahman B, Mark T (2010). Subsurface fracture analysis and determination of in-situ stress direction using FMI logs: An example from the Santonian carbonates (Ilam Formation) in the Abadan Plain. Iran. Tectonophys..

[2] Tan YS, Li Q, Xu L (2022). A critical review of carbon dioxide enhanced oil recovery in carbonate reservoirs. Fuel.

[3] Yudong, W. *Natural Gas Development and Utilization* (China Petrochemical Press, 2011).

[4] Luo Z, Cao Q, Yang HJ (2021). Drilling design for UGSs rebuilt from fractured gas reservoirs: An example from Tongluoxia UGS. Nat. Gas Explor. Dev..

[5] Milad B, Slatt R (2018). Impact of lithofacies variations and structural changes on natural fracture distributions. Interpretation.

[6] Nelson, R. *Geologic Analysis of Naturally Fractured Reservoirs* (Elsevier, 2001).

[7] Xu S, Gou QY, Hao F (2020). Multiscale faults and fractures characterization and their effects on shale gas accumulation in the Jiaoshiba area, Sichuan Basin, China. J. Geophys. Eng..

[8] Zhang SL, Yan JP, Cai JG (2021). Fracture characteristics and logging identification of lacustrine shale in the Jiyang Depression, Bohai Bay Basin, Eastern China. Mar. Pet. Geol..

[9] Dong SQ, Wang ZZ, Zeng LB (2016). Lithology identification using kernel Fisher discriminant analysis with well logs. J. Petrol. Sci. Eng..

[10] Tokhmchi B, Memarian H, Noubari HA, Moshiri B (2009). A novel approach proposed for fractured zone detection using petrophysical logs. J. Geophys. Eng..

[11] Sun JM, Liu R, Mei JX (1999). Fracture Identification Technique for Conventional Logs from Western Chaidamu Basin. Qinghai Oilfield. WLT.

[12] Lin LF, Gao Y, Yin S (2022). Log evaluation for tight oil reservoir of Yanchang Formation in western oil region of Ordos Basin. Logging Technology.

[13] He L (2010). Evaluation of fracture-porosity carbonate reservoirs with conventional logging data. J. Pet. Nat. Gas.

[14] Chen D, Wei XC (2010). Well-logging evaluate technology for fractured carbonate reservoir in Tahe area. GPP.

[15] Lyu WY, Zeng LB, Liu ZQ (2016). Fracture responses of conventional logs in tight-oil sandstones: a case study of the Upper Triassic Yanchang Formation in southwest Ordos Basin, China. AAPG Bull..

[16] Ja’Fari A, Kadkhodaie-Ilkhchi A, Sharghi Y (2011). Ghanavati. Fracture density estimation from petrophysical log data using the adaptive neuro-fuzzy inference system. J. Geophys. Eng..

[17] Zazoun RS (2013). Fracture density estimation from core and conventional well logs data using artificial neural networks: The Cambro-Ordovician reservoir of Mesdar oil field, Algeria. J. Afr. Earth Sci..

[18] Dong SQ, Zeng LB, Che XH (2023). Application of artificial intelligence in fracture identification using well logs in tight reservoirs. Geosciences.

[19] Du CZ, Duan YX, Sun QF (2021). Seismic fault identification method based on ResUNet and Dense CRF model. J. Appl. Sci..

[20] Geng HJ, Wang GW, Li J (2002). Research center for imaging logging image interpretation modes and typical interpretation charts. J. Jianghan Pet..

[21] Fan H, Shi JY, Fan TL (2021). Sedimentary microfacies analysis of carbonate formation based on FMI and conventional logs: A case study from the ordovician in the Tahe Oilfield, Tarim Basin, China. J. Pet. Sci. Eng..

[22] Chitale VD, Johnson C, Entzminger D, Canter L (2010). Application of a modern electrical borehole imager and a new image interpretation technique to evaluate the porosity and permeability in carbonate reservoirs: a case history from the permian basin, United States. AAPG Memoir.

[23] Liu SJ (2003). Application of FMI imaging logging in fracture reservoir evaluation of Chegu 20 buried hill. Oil Gas Geophys..

[24] Tong HM (2006). Application of imaging well logging data in prediction of structural fracture. Nat. Gas Ind..

[25] Fan AP, Yang RC, Lenhardt N (2019). Cementation and porosity evolution of tight sandstone reservoirs in the Permian Sulige gas field, Ordos Basin (central China). Mar. Pet. Geol..

[26] Xie K, Tan XC, Feng M (2020). Eogenetic karst and its control on reservoirs in the Ordovician Majiagou Formation, eastern Sulige gas field, Ordos Basin, NW China. Pet. Explor. Dev..

[27] Li JB, Wu XN, Zhao ZJ (2016). Characteristics and origin of upper Paleozoic fractures in Sulige Gas Field. J. Xi'an Univ. Sci. Technol..

[28] Ji MM, Zhao JX, Li FJ (2013). Diagenesis and pore evolution of carbonate rock in Ordovician Mawu5 submember, eastern Sulige area. Nat. Gas Explor. Dev..

[29] Zhang YB (1965). Microscopic study of fractures and holes. Pet. Geol. Experim..

[30] Wu HY, Zhao JZ, Wu WT (2021). Formation and diagenetic characteristics of tight sandstones in closed to semi-closed systems: Typical example from the Permian Sulige gas field. J. Pet. Sci. Eng..

